# Acetyl Salicylic Acid, COX‐2 Inhibitors and Other NSAIDs and Breast Cancer Survival in a Finnish Population‐Based Cohort

**DOI:** 10.1002/cnr2.70271

**Published:** 2025-07-05

**Authors:** M. Malin, M. Murto, O. Arponen, A. Jukkola, A. Siltari, M. Artama, K. Visvanathan, T. Murtola

**Affiliations:** ^1^ Tampere University Faculty of Medicine and Health Technology Tampere Finland; ^2^ Department of General Surgery TAYS Cancer Center Tampere Finland; ^3^ Department of Oncology TAYS Cancer Center Tampere Finland; ^4^ Department of Pharmacology Faculty of Medicine, University of Helsinki Helsinki Finland; ^5^ Department of Health Protection Finnish Institute for Health and Welfare Tampere Finland; ^6^ Department of Epidemiology Johns Hopkins Bloomberg School of Public Health Baltimore Maryland USA; ^7^ Department of Oncology Johns Hopkins School of Medicine Baltimore Maryland USA; ^8^ Department of Urology TAYS Cancer Center Tampere Finland

## Abstract

**Background:**

Non‐steroidal anti‐inflammatory drugs (NSAIDs), particularly acetylsalicylic acid (ASA), have been associated with reduced breast cancer (BCa) mortality. While overexpression of cyclooxygenase‐2 (COX‐2) correlates with poorer prognosis in BCa, COX‐2 inhibitors (coxibs) have not demonstrated a survival advantage. However, the evidence remains conflicting and limited. We examined associations between BCa mortality and NSAID use in a Finnish population‐based cohort.

**Methods:**

The study cohort, 73 170 women with new BCa diagnosis during 1995–2013 was identified from The Finnish Cancer Registry. Follow‐up data including date and the cause of death, NSAID purchases from 1995 to 2015, mammography screening participation and tumor hormone receptor status, were obtained from national registries. NSAID purchases were categorized into NSAIDs overall, ASA, and coxibs. BCa‐specific and overall survival by NSAID use were analyzed using Cox proportional hazard regression, adjusted for age, tumor extent, primary treatment, Charlson comorbidity index, hypertension, diabetes, mammography participation, and hormonal therapy.

**Results:**

Pre‐diagnostic use of NSAIDs (HR 0.78, 95% CI: 0.75–0.81) and coxibs (HR 0.76, 95% CI: 0.71–0.81) was associated with reduced BCa mortality, while ASA showed no association. Post‐diagnostic NSAID use was associated with increased BCa mortality (HR 1.27, 95% CI: 1.22–1.33), while ASA use (HR 0.84, 95% CI: 0.73–0.97) showed dose‐dependent risk reduction.

**Conclusion:**

Post‐diagnostic use of ASA is associated with reduced BCa‐specific mortality, distinguishing ASA from other NSAIDs. Clinical trials are required to determine the ideal ASA dose, frequency, and duration for treating BCa. Pre‐diagnostic use of NSAIDs overall is associated with a slight reduction in BCa mortality without dose dependence. The potential role of pre‐diagnostic NSAID use as a prognostic factor in BCa warrants further investigation.

## 1 Introduction

With 2.3 million new cases and 666 000 deaths in 2022, breast cancer (BCa) is the most common cancer globally and one of the leading causes of death among females [1]. BCa is a heterogeneous disease with a variable prognosis. Several risk factors for BCa, including age, obesity, BRCA mutations, and hormonal factors, are well established [2]. Chronic inflammation has been associated with a risk of developing BCa [3]. It has been suggested that chronic inflammation may facilitate carcinogenesis through various mechanisms, such as enhanced cell proliferation, evasion of apoptosis, increased angiogenesis, and the promotion of metastasis formation [4].

Non‐steroidal anti‐inflammatory drugs (NSAIDs) are among the most widely prescribed medications, accounting for approximately 5% of all prescriptions globally [5]. NSAIDs inhibit cyclooxygenase enzymes (COXs), which play a key role in the synthesis of prostanoids that are crucial for initiating the inflammatory response [6]. Classification of NSAIDs is based on their prostaglandin synthase selectivity between cyclooxygenase isoenzyme types (COX‐1 and COX‐2). COX‐1 is constitutively expressed in various tissues, whereas COX‐2 expression is upregulated during acute inflammatory responses. Non‐selective NSAIDs inhibit both COX‐1 and COX‐2 forms with varying degrees of specificity, while coxibs selectively target COX‐2. The mechanism of action of acetylsalicylic acid (ASA) distinguishes it from other NSAIDs, as it is a non‐competitive and irreversible inhibitor of both COX‐1 and COX‐2, exhibiting greater affinity for the COX‐1 isoform [7].

Previous studies have demonstrated an association between post‐diagnostic NSAID use and reduced cancer‐specific mortality [8]. This evidence has been particularly clear in gastrointestinal tract cancers and ASA use. Additionally, ASA use has been linked to reduced cancer‐specific mortality and reduced risk of hormone receptor‐positive tumors in BCa [9, 10]. Post‐diagnostic use of other NSAIDs has shown some similar protective associations on BCa survival [11]. However, findings have been partially contradictory, and the amount of evidence remains relatively limited. Celecoxib, a coxib‐class drug, has demonstrated antitumoral effects in preclinical models of human BCa. Certain malignancies, including BCa, exhibit overexpression of COX‐2 compared to adjacent healthy tissue [12]. This overexpression has been associated with poor prognosis, predicting lymph node metastasis and larger tumor size. Elevated COX‐2 expression in BCa may promote tumor angiogenesis, cell proliferation, invasiveness, metastasis and apoptosis inhibition [13]. However, despite the mechanistic alignment between coxibs' mechanism of action and the overexpression of COX‐2 in BCa, there is no definitive clinical evidence to support the use of coxibs as a therapeutic option for BCa in humans [14, 15].

NSAIDs are used to treat cancer related pain. A standard clinical practice in Finland is to initiate analgesic use with NSAIDs and acetaminophen. These drugs stay in use but are often supplemented with opioids in later stages of cancer [16]. Therefore, the intensity and duration of NSAID use are often substantial in patients with extensive symptomatic metastases. This should be taken into consideration when epidemiologically evaluating the association between BCa mortality and NSAID use.

The objective of this study was to evaluate the association between NSAID use and BCa mortality at the population level in a Finnish cohort of female patients with BCa. We aimed to control the potential biases mentioned above in order to estimate the direct effects of NSAID, ASA and coxib use. In addition, we investigated the potential influence of hormone receptor status.

## 2 Materials and Methods

### 2.1 Study Population

The study cohort was formed by identifying all newly diagnosed BCa cases between 1995 and 2013 from The Finnish Cancer Registry. It has been mandatory in Finland for health care practitioners to report new cancers to the Finnish Cancer Registry since 1960s and it therefore covers practically all cancer diagnoses made in the Finnish health care units [17]. Additional information on tumor histology, time of the BCa diagnosis, information on primary treatment types (surgery vs. other treatments) and data on tumor extent (from 91.3% of the cases) were also available. Follow‐up data, including the date and the cause of death was available until the end of 2015 from the Causes of Death register of Statistics Finland [18]. The Finnish Mass Screening Registry provided the data of the individual's participation in mammography screening during the study period.

The Finnish breast cancer screening program consists of personal invitations sent every 2 years. Mammography is the primary screening method. If needed, further assessments including ultrasound and biopsy are carried out. Target population involves all Finnish women aged 50–69 years old. The invitational coverage remains at nearly 100%. In 2015 83% of the invited women participated to screening. Data on screening are recorded in electronic format by the Finnish Mass Screening Registry [19]. Information on tumor hormone receptor status was available from the leading regional providers of laboratory services in three largest metropolitan areas in Finland: Helsinki, Tampere and Turku. Data on hormone receptor status (ER, PR, HER2) was available for a subpopulation of 11 179 (15.3%) female.

### 2.2 NSAID Purchases and Dose Standardization

In Finland, NSAIDs can be purchased both with a prescription and over the counter, with the exception of coxibs which are available only by prescription. The information on physician‐prescribed NSAIDs purchased along with purchase dates, total amount, and dose during the study period between 1995 and 2015 were collected for the study cohort from the national Prescription Register, administered by the governmental Social Insurance Institution of Finland (SII). The SII reimburses drug purchases if the medication has been prescribed by a physician and approved as reimbursable by the SII [20]. The reimbursement data was then linked to the study population using personal identification code. Data on prescription‐free over‐the‐counter purchases and hospital in‐patient drug use were unavailable, as such information is not recorded in Finland in a way that permits linkage to individual users. Dose variations between different NSAIDs were standardized by dividing the total milligram dose of participants annual purchases of each NSAIDs with the drug specific Defined Daily Dosages (DDDs) listed by the World Health Organization [21].

Separate variables were created for all NSAIDs combined and separately for ASA, coxibs and purchases of non‐ASA, non‐coxib NSAIDs (Table S1). NSAID user status was determined for each year separately. Participants were classified as “non‐users” for years without recorded NSAID purchases. For years with any recorded NSAID, ASA or coxib purchases, participants were classified as “users.” NSAID use before and after BCa diagnosis was analyzed separately. In the pre‐diagnostic analysis, user status was categorized as “pre‐diagnostic user,” if any NSAID use had been recorded between 1995 and the year of BCa diagnosis. All pre‐diagnostic use since 1995 was included regardless of the duration or amount of use. For post‐diagnostic NSAID use, the user status and the duration of usage were updated cumulatively for each follow‐up year based on the recorded purchases. These were used to generate time‐dependent variables for NSAID use, which was updated prospectively for each year of the follow‐up period. Intensity of NSAID use was estimated by dividing the cumulative DDD amount of each follow‐up year by the number of years with recorded NSAID purchases at that year.

The Care Register for Health Care is a national registry with a purpose of collecting data on the activities of health centers, hospitals and other institutions providing inpatient care for the purposes of statistics, research and planning [22]. This registry was used to obtain information on diagnosis recorded during in‐ and outpatient hospital visits within the study period. International Statistical Classification of Diseases and Related Health Problems 10th Revision (ICD‐10) codes recorded in the registry were used to calculate the Charlson comorbidity index for each study participant.

Ethical approval was not obtained from this study as it is based solely on routinely recorded national registry data. The study did not affect patient care or involve direct patient contact at any phase. Therefore, as per Finnish legislature, separate ethics board approval was not required, but the study protocol was reviewed and approved by the keepers of registry databases before the data was obtained (26.4.2019/552 Law on secondary use of health care data, www.finlex.fi/en).

### 2.3 Statistical Analysis

The Cox regression models were used to analyze the correlations between pre‐ and post‐diagnostic NSAID use and BCa‐specific and overall deaths by calculating hazard ratios (HR) and 95% confidence intervals (CIs). The NSAID non‐users were used as the reference group in all analyses. The risk of BCa‐specific and overall mortality were analyzed for combined NSAID use, and separately for ASA and coxibs use to appreciate their different mechanisms of action.

The follow‐up began at the date of BCa diagnosis and ended at death, emigration or at the common closing date December 31, 2015, whichever came first. NSAID use before and after BCa diagnosis was analyzed separately. In the pre‐diagnostic analysis, user status was categorized as “pre‐diagnostic user,” if any NSAID use had been recorded between 1995 and the year of BCa diagnosis. All pre‐diagnostic use since 1995 was included regardless of the duration or amount of use. For post‐diagnostic NSAID use, the user status and the duration of usage were updated cumulatively for each follow‐up year based on the recorded purchases. These were used to generate time‐dependent variables for NSAID use, which was updated prospectively for each year of the follow‐up period. Intensity of NSAID use was estimated by dividing the cumulative DDD amount of each follow‐up year by the number of years with recorded NSAID purchases at that year. Analyses were adjusted for age at the time of BCa diagnosis. Multivariate analyses were additionally adjusted for tumor extent, Charlson comorbidity index, primary treatment method (surgery or other), use of anti‐hypertensive or anti‐diabetic drugs, participation in mammography screening and the possible use of hormonal therapy.

We performed lag time analyses to reduce protopathic bias, a method recommended in pharmacoepidemiologic studies [23]. We evaluated the associations between NSAID use and BCa deaths with varying time lags. For example, when the 3‐year lag time analysis was performed, we studied BCa deaths occurring 3 years after the NSAID use, censoring the last 3 years of use. The lag time analysis removes the effect of changes to the user status during the final years before death.

Subgroup analyses were stratified by participants hormone receptor statuses grouped as being positive or negative for ER, PR, and HER2, respectively. The analyses of post‐diagnostic use were repeated within these subgroups. We performed a sensitivity analysis limited to non‐ASA and non‐coxib NSAIDs. Additionally, NSAIDs, ASA and coxibs were analyzed as risk factors for BCa death in competing risks regression analyses with non‐cancer deaths as the competing risk.

Statistical analyses were performed using IBM SPSS 25 (Chicago, IL, USA) Competing risks regression analyses were performed using Stata 17 software (College Station, TX, USA).

## 3 Results

### 3.1 Population Characteristics

The study cohort consisted of 73 170 women (Table 1). Of these, 48 124 (65.8%) had any NSAID purchases before the BCa diagnosis, while 55 974 (76.5%) used NSAIDs after the diagnosis. Participants who had purchased NSAIDs before or after BCa diagnosis had lower crude BCa and overall mortality than participants with no use. Participants who had purchased NSAIDs after BCa diagnosis tended to be younger (median age 59 vs. 71 years at diagnosis). Similar differences in participant age between users and nonusers were not observed for pre‐diagnostic use (Table 1).

**TABLE 1:** Characteristics of the study cohort of 73 170 female BCa patients diagnosed in Finland during the years 1995–2013 by the pre‐ and post‐diagnostic NSAID user status.

Characteristic	NSAID use before BCa diagnosis		NSAID use after BCa diagnosis	
	No	Yes	No	Yes
Total participants (*n*)	25 046	48 124	17 196	55 974
Median follow‐up time (years, IQR)	8.2 (3.5–13.6)	4.9 (2.2–8.9)	3.1 (1.2–6.7)	6.8 (3.2–11.3)
*N* of BC deaths (%)	5184 (20.7%)	5716 (11.9%)	3461 (20.1%)	7439 (13.3%)
BC mortality (/1000 person years)	342/1000	161/1000	388/1000	178/1000
Overall deaths (%)	9878 (39.4%)	12 642 (26.3%)	8270 (48.1%)	14 250 (25.5%)
Overall mortality (/1000 person years)	651/1000	356/1000	927/1000	342/1000
Median age at diagnosis	60	62	71	59
(IQR)	(50–71)	(54–72)	(61–81)	(51–68)
Tumor stage at diagnosis				
Localized (%)	12 312 (49.2%)	24 324 (50.5%)	7604 (44.2%)	29 032 (51.9%)
Locally advanced (%)	8243 (32.9%)	15 800 (32.8%)	5017 (29.2%)	19 026 (34.0%)
Metastatic (%)	2235 (8.9%)	3890 (8.1%)	2367 (13.8%)	3758 (6.7%)
Unknown (%)	2256 (9.0%)	4110 (8.5%)	2208 (12.8%)	4158 (7.4%)
Tumor morphology				
Invasive ductal (%)	19 096 (76.2%)	36 775 (76.4%)	12 459 (72.5%)	43 412 (77.6%)
Invasive lobular (%)	4107 (16.4%)	8198 (17.0%)	2785 (16.2%)	9520 (17.0%)
Other (%)	1843 (7.4%)	3151 (6.5%)	1952 (11.4%)	3042 (5.4%)
Hormone receptor status				
ER + (% of cases with information available)	2490 (86.3%)	8689 (87.5%)	2551 (87.2%)	8628 (87.2%)
ER − (% of cases with information available)	396 (13.7%)	1245 (12.5%)	376 (12.8%)	1265 (12.8%)
*ER status unknown*	22 160	38 190	14 269	46 081
PR + (% of cases with information available)	1941 (67.9%)	6955 (70.5%)	2036 (70.0%)	6860 (69.9%)
PR − (% of cases with information available)	919 (32.1%)	2911 (29.5%)	873 (30.0%)	2957 (30.1%)
*PR status unknown*	22 168	38 258	14 287	46 157
HER2 + (% of cases with information available)	785 (26.3%)	2962 (30.1%)	800 (27.5%)	2947 (29.8%)
HER2 − (%of cases with information available)	2200 (73.7%)	6870 (69.9%)	2112 (72.5%)	6958 (70.2%)
*HER2 status unknown*	22 061	38 292	14 284	46 069
Mammography screening history				
None	12 023 (48.0%)	18 757 (39.0%)	9821 (57.1%)	20 959 (37.4%)
Any	13 025 (52.0%)	29 338 (61.0%)	7379 (42.9%)	35 081 (62.6%)
Primary treatment‐method				
Curative‐treatment surgery	17 172 (68.6%)	31 659 (65.8%)	9820 (57.1%)	39 011 (69.7%)
Other/unknown	7874 (31.4%)	16 465 (34.2%)	7376 (42.9%)	16 963 (30.3%)
Antihormonal therapy				
Any	9565 (38.2%)	18 099 (37.6%)	4586 (26.7%)	23 078 (41.2%)
Comorbiditiies				
Charlson comorbidity index mean (IQR)	0 (0–0)	0 (0–1)	0 (0–1)	0 (0–1)
Hypertension	16 020 (64.0%)	34 849 (72.4%)	12 535 (72.9%)	38 334 (68.5%)
Diabetes mellitus	3561 (14.2%)	8115 (16.9%)	3194 (18.6%)	8482 (15.2%)

No significant differences were observed in disease extent at diagnosis in relation to pre‐diagnostic medication use. Patients with NSAID purchases after BCa diagnosis had less metastatic cancer at the time of diagnosis than non‐users (6.7% vs. 13.8%) (Table 1).

Participants who had used NSAIDs before or after BCa diagnosis had more often attended mammography screenings compared to non‐users. Post‐diagnostic NSAID users also used hormone therapy more often compared to the ones with no post‐diagnostic NSAID use. Similar differences were not observed among pre‐diagnostic NSAID users (Table 1).

No significant differences were observed in other examined characteristics, such as tumor histology, primary treatment methods, Charlson Comorbidity Index or other comorbidities depending on NSAID use (Table 1).

### 3.2 Pre‐Diagnostic NSAID Use and Cancer Specific Survival

Pre‐diagnostic use of any NSAID (HR 0.78, 95% CI: 0.75–0.81) or coxibs (0.76 95% CI: 0.71–0.81) was associated with lower risk of BCa mortality compared to non‐users after multivariable adjustment (Figure S1). The association did not depend on yearly dose of medication use (Table 2).

**TABLE 2:** The risk of breast cancer specific death among pre‐diagnostic users and non‐users of NSAIDs in a cohort of 73 170 females diagnosed with breast cancer in Finland 1995–2013.

	NSAIDs overall			ASA			Coxibs		
	*n* of cases/deaths	HR (95% CI)_age‐adjusted_	HR (95% CI)_multivar_._‐adjusted_ ^a^	*n* of cases/deaths	HR (95% CI)_age‐adjusted_	HR (95% CI)_multivar_._‐adjusted_ ^a^	*n* of cases/deaths	HR (95% CI)_age‐adjusted_	HR (95% CI)_multivar_._‐adjusted_ ^a^
Medication use									
None	17 196/3461	Ref	Ref	71 428/10 619	Ref	Ref	60 322/9908	Ref	Ref
Any	55 974/7439	0.71 (0.69–0.74)	0.78 (0.75–0.81)	1743/281	0.97 (0.86–1.01)	0.92 (0.81–1.03)	12 848/992	0.68 (0.64–0.73)	0.76 (0.71–0.81)
Average doses per year									
1st tertile^a^	16 041/1804	0.68 (0.64–0.71)	0.74 (0.71–0.79)	596/85	0.93 (0.75–1.15)	0.90 (0.73–1.11)	4452/335	0.66 (0.59–0.74)	0.75 (0.67–0.83)
2nd tertile^b^	16 048/1704	0.67 (0.64–0.71)	0.75 (0.71–0.79)	582/82	0.94 (0.75–1.16)	0.88 (0.71–1.10)	4412/295	0.92 (0.55–0.69)	0.68 (0.60–0.76)
3rd tertile^b^	16 035/2208	0.79 (0.75–0.83)	0.85 (0.81–0.89)	565/114	1.02 (0.85–1.23)	0.96 (0.80–1.15)	3984/362	0.77 (0.70–0.86)	0.85 (0.77–0.95)

^a^ Calculated with Cox regression with model adjustment for age at diagnosis, Charlson comorbidity index, primary treatment method (surgery or other), use of anti‐hypertensive or anti‐diabetic drugs, participation in mammography screening, and the possible use of hormonal therapy.

^b^ Tertile ranges: NSAIDs: 1st tertile 0.1–26.2 DDD/year, 2nd tertile 26.3–55 DDD/year, 3rd tertile > 55 DDD/year, ASA: 1st tertile 0.1–200 DDD/year, 2nd tertile 200.1–400 DDD/year, 3rd tertile > 400 DDD/year, coxibs: 1st tertile 0.1–20 DDD/year, 2nd tertile 20.1–42 DDD/year, 3rd tertile > 42 DDD/year.

Pre‐diagnostic use of ASA did not significantly associate with BCa mortality even at high doses (more than 400 DDD/year) compared to nonuse (HR 0.92, 95% CI: 0.81–1.03) (Table 2).

### 3.3 Post‐Diagnostic NSAID Use and Cancer‐Specific Survival

Post‐diagnostic use of NSAIDs was associated with significantly elevated BCa mortality compared to non‐users in multivariable adjusted analysis (HR 1.27, 95% CI: 1.22–1.33) (Table 3, Figure S1). A weaker risk increase was also observed for coxibs (HR 1.10, 95% CI: 1.04–1.16). The use of NSAIDs and coxibs increased the risk of BCa mortality in a dose‐dependent manner. In contrast, post‐diagnostic ASA use associated with lower BCa‐specific mortality (HR 0.84, 95% CI: 0.73–0.97) compared to non‐users. The risk association for ASA use and breast cancer mortality was inverse, i.e., the higher the average yearly dose, the lower the breast cancer‐specific mortality was (Table 3).

**TABLE 3:** The risk of breast cancer specific death among post‐diagnostic users and non‐users of NSAID in a cohort of 73 170 females diagnosed with breast cancer in Finland 1995–2013.

	NSAIDs overall			ASA			Coxibs		
	*n* of deaths/cases	HR (95% CI)_age‐adjusted_	HR (95% CI)_multivar_._‐adjusted_ ^a^	*n* of deaths/cases	HR (95% CI)_age‐adjusted_	HR (95% CI)_multivar_._‐adjusted_ ^a^	*n* of deaths/cases	HR (95% CI)_age‐adjusted_	HR (95% CI)_multivar_._‐adjusted_ ^a^
Medication use									
None	3558/17 631	Ref	Ref	10 693/71 032	Ref	Ref	9629/58 162	Ref	Ref
Any	7342/55 539	1.22 (1.17–1.28)	1.27 (1.22–1.33)	207/2138	0.83 (0.72–0.95)	0.84 (0.73–0.97)	1271/15 008	1.02 (0.97–1.07)	1.10 (1.04–1.16)
Average doses per year									
1st^b^ tertile	1746/17 262	0.79 (0.75–0.83)	0.87 (0.82–0.92)	67/706	0.95 (0.74–1.22)	0.94 (0.74–1.21)	508/6231	0.90 (0.83–0.97)	0.99 (0.91–1.06)
2nd^b^ tertile	2014/18 521	1.17 (1.11–1.24)	1.22 (1.15–1.29)	82/718	0.97 (0.76–1.22)	0.94 (0.75–1.19)	284/3833	1.01 (0.91–1.12)	1.12 (1.00–1.23)
3rd^b^ tertile	3582/19 756	1.76 (1.67–1.84)	1.69 (1.61–1.77)	58/714	0.70 (0.55–0.89)	0.74 (0.58–0.94)	479/4944	1.20 (1.11–1.29)	1.25 (1.15–1.35)

^a^ Calculated with Cox regression with model adjustment for age at diagnosis, Charlson comorbidity index, primary treatment method (surgery or other), use of anti‐hypertensive or anti‐diabetic drugs, participation in mammography screening, and the possible use of hormonal therapy.

^b^ Tertile ranges: NSAIDs: 1st tertile 0.1–33 DDD/year, 2nd tertile 33.1–66 DDD/year, 3rd tertile > 66 DDD/year, ASA: 1st tertile 0.1–183 DDD/year, 2nd tertile 183.1–314 DDD/year, 3rd tertile > 314 DDD/year, coxibs: 1st tertile 0.1–28 DDD/year, 2nd tertile 28.1–50 DDD/year, 3rd tertile > 50 DDD/year.

### 3.4 Lag Time Analysis of Post‐Diagnostic Use of NSAIDs

Post‐diagnostic use of any NSAIDs continued to be significantly associated with elevated BCa mortality even after 3 years' time lag; the risk difference attenuated close to null but did not entirely disappear (Table 4). The inverse association between ASA use and BCa‐specific mortality disappeared with 1‐year time lag.

**TABLE 4:** Risk of breast cancer death by NSAID use before diagnosis with 1–3 year time lag. Cohort of 73 170 women diagnosed with breast cancer in Finland 1995–2013.

	NSAIDs overall				ASA			
	*n* of deaths/cases	HR (95% CI)_multivar_._‐ adjusted_ ^a^	1‐year time lag	3‐year time lag	*n* of deaths/cases	HR (95% CI) _multivar_._‐adjusted_ ^a^	1‐year time lag	3‐year time lag
None	3558/17 631	Ref	Ref	Ref	10 693/71 032	Ref	Ref	Ref
Any	7342/55 539	1.27 (1.22–1.33)	1.19 (1.14–1.24)	1.06 (1.02–1.11)	207/2138	0.84 (0.73–0.97)	0.91 (0.79–1.05)	0.99 (0.84–1.16)

^a^ Calculated with Cox regression with model adjustment for age at diagnosis, Charlson Comorbidity Index, primary treatment method (surgery or other), use of anti‐hypertensive or anti‐diabetic drugs, participation in mammography screening, and the possible use of hormonal therapy.

### 3.5 NSAID Use and Overall Mortality

The effect of pre‐ and post‐diagnostic NSAID, coxib and ASA use on overall mortality are shown in Tables S1 and S2. Pre‐diagnostic use of NSAIDs (HR 0.93 95% CI: 0.91–0.96) and coxibs (HR 0.94 95% CI: 0.90–0.98) were associated with marginally lower risk of overall mortality compared to non‐users. Post‐diagnostic use of NSAIDs or coxibs did not associate with overall mortality (Tables S2 and S3). ASA use, both before and after BCa diagnosis, was associated with higher overall mortality.

### 3.6 Subgroup Analyses

**FIGURE 1:**
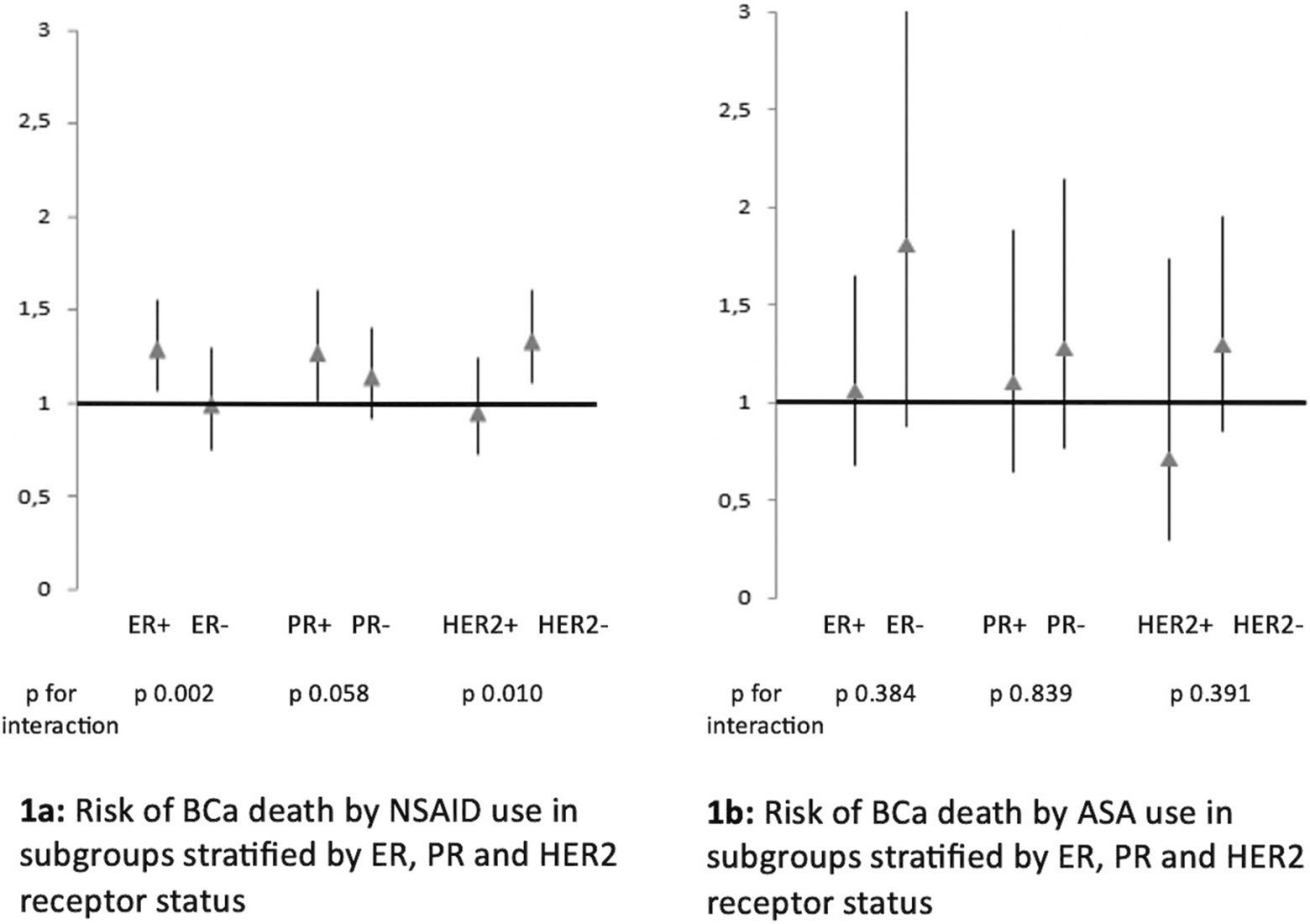
(a, b) The risk of dying of breast cancer by NSAID and ASA use in subgroups stratified by ER, PR and HER2 receptor statuses in a cohort of 11 179 females diagnosed with breast cancer in Finland 1995–2013 whose hormone receptor status data was available. (b) Risk of BCa death by ASA use in subgroups stratified by ER, PR and HER2 receptor status, (a) Risk of BCa death by NSAID use in subgroups stratified by ER, PR and HER2 receptor status.

ER‐ and HER2‐receptor status modified the risk association between post‐diagnostic NSAID use and BCa‐specific mortality (Figure 1). The risk was elevated among NSAID users compared to non‐users in the subgroups of patients with ER‐positive and HER2‐negative tumors (*p* for interaction 0.002 and 0.010 for ER and HER2, respectively). In the subgroup of PR‐positive tumors, post‐diagnostic NSAID use was similarly associated with increased risk of BCa mortality. However, the interaction by PR status was only approaching statistical significance (*p* for interaction 0.058).

In the subgroup with hormone receptor status information available, ASA use was generally not associated with BCa‐specific mortality. However, the risk association between ASA use and BCa mortality was not significantly modified by hormone receptor status (Figure 1). The number of coxib users was too low for subgroup analyses.

### 3.7 Sensitivity Analysis

In a sensitivity analysis excluding ASA and coxib users, pre‐diagnostic NSAID use continued to be associated with lowered risk of BCa death compared to non‐users (HR 0.78, 95% CI: 0.75–0.81). Pre‐diagnostic NSAID use was associated with lower BCa mortality also in competing risks analysis (HR 0.77, 95% CI: 0.74–0.80), confirming that the protective cancer association is not due to increased risk of dying from non‐cancer causes among NSAID users. Similarly, pre‐diagnostic use of coxibs (HR 0.72 95% CI: 0.67–0.77) and ASA (HR 0.79 95% CI: 0.69–0.90) were associated with lowered BCa mortality in competing risks regression analyses.

## 4 Discussion

This population‐based study of 73 170 female patients diagnosed with BCa in Finland between 1995 and 2013 showed a short‐term post‐diagnostic use of ASA associated with reduced BCa‐specific mortality, whereas post‐diagnostic NSAID use associated with a short‐term risk increase. On the other hand, pre‐diagnostic use of any NSAIDs and coxibs were associated with lower risk of BCa death compared to non‐users even after multivariable adjustment with no dose dependency. Pre‐diagnostic use of ASA did not significantly associate with BCa mortality even at high doses. Pre‐ and post‐diagnostic ASA use was associated with higher overall mortality which might be due to prescription ASA being used mainly for secondary prevention of stroke and other cardiovascular incidences. NSAIDs combined or coxibs separately did not have any dose‐dependent association with overall mortality before or after the BCa diagnosis.

The observed reduction in BCa‐specific mortality with post‐diagnostic ASA use is coherent with previous research results [8, 9]. In previous studies, ASA use after diagnosis has been associated with reduced cancer‐specific mortality in multiple cancer types, especially in cancers of the GI‐tract [8]. Similar protective associations have also been observed between ASA use and BCa‐specific mortality [9].

BCa is a highly heterogeneous disease, and different subtypes significantly differ in terms of prognosis and treatment modalities, which impacts the interpretation of the results. Particular attention should be given to hormone receptor status and HER2 positivity, which play a key role in classifying cases into different biological subgroups. Since NSAIDs do not exert hormonal effects, it could naturally be assumed that hormone receptor status would not significantly influence the outcomes. In line with this, we did not observe in this study any clear differences in the associations between NSAID use and the risk of BCa mortality across biological subgroups defined by hormone receptor status or HER2 status. However, it is important to note this information was only available for a limited subset of cases in the study population.

To our knowledge, previous studies have not estimated the risk of BCa‐specific mortality among ASA users by hormone receptor status. However, BCa risk has been reported to be lowered among ASA users for hormone receptor positive, but not negative tumors [10].

ASA differs from other NSAIDs in two important aspects; first, ASA is not commonly used to treat cancer‐specific pain. Thus, protopathic bias due to advanced disease is less likely. Instead, ASA is commonly used in primary and secondary prevention of cardiovascular events, which may cause a different type of selection bias, as ASA users may be in elevated risk of dying of non‐cancer causes compared to non‐users. However, the risk decrease persisted even in competing risks analysis that took into account non‐cancer mortality. Second, ASA's mechanism of action differs from other NSAIDs as it is the only non‐competitive and irreversible inhibitor of COX‐1 and COX‐2 receptors [24]. Therefore, inhibition of prostaglandin production might be more powerful with ASA than other NSAIDs.

For ASA, the dose‐dependence was inverse; the risk of BCa‐specific death was further lowered by increasing yearly dose of post‐diagnostic ASA use. This suggests the risk reduction may be caused by ASA use. Our lag time analysis suggests this risk association is short‐term as it nearly disappears already with 1 year time‐lag. Lag time analysis does reduce the effect of protopathic bias which suggests the findings to be either due such bias or that the biological effect of ASA is short‐term, which is plausible given that platelet function recovers in 7–10 days (platelet life span) after cessation of ASA use [24]. Given that ASA has shown such promising results in terms of its anticancer effects, as observed in this and previous studies, further research on this topic is needed. In particular, future studies should focus on enhancing, targeting, and optimizing its anticancer effects, for example, by improving drug delivery to cancer cells through the use of nanoparticles [25].

Post‐diagnostic use of NSAIDs combined and coxibs was associated with increased BCa‐specific mortality compared with non‐users. Some previous studies have reported contradictory results, as post‐diagnostic non‐ASA NSAID use has shown a similar protective association with BCa survival as ASA [11]. This might be explained by the fact that it is common practice in Finland to use non‐ASA NSAIDs as part of the treatment of cancer‐related pain [16]. This causes protopathic bias, as patients with advanced disease are more likely to use non‐ASA NSAIDs with a prescription compared to those with localized disease, and this must be taken into consideration when interpreting these findings.

The association between post‐diagnostic NSAID use and elevated BCa mortality was dose‐dependent, i.e., post‐diagnostic NSAIDs use associated with increased BCa mortality. Two possible explanations may be behind the dose‐dependency. Either NSAIDs indeed cause BCa progression, or protopathic bias is strongest with high‐dose NSAID use. It is logical to assume that cancer patients with the greatest metastatic burden and most pain would use NSAID more than patients with localized disease. Also, locally advanced BCa may have been treated with more extensive surgery, which would cause more pain afterwards and require greater amounts of NSAID use. Lag‐time analyses support the role of protopathic bias as the risk elevation attenuates with increasing time lag.

We did not observe any risk reduction by post‐diagnostic use of COX‐2 selective coxibs. The results for coxib use were similar to NSAIDs combined, although the risk increase was more marginal. In contrast, pre‐diagnostic coxib and NSAID use were associated with a slight risk decrease in a non‐dose‐dependent manner. Sensitivity analysis showed this not to be caused by coxibs only, as the risk reduction persisted after exclusion of coxibs and ASA. This raises the question of whether NSAIDs could have beneficial effects on BCa prognosis when used before the diagnosis. COX‐2 overexpression is associated with poor BCa prognosis [12]. This has aroused interest in the possibilities of COX‐2 inhibition in the treatment of BCa. Despite this compatibility, so far, clinical trials with coxibs have not shown clear results supporting the hypothesis of COX‐2 inhibition being beneficial in BCa [14, 15]. Our results partly comply with these previous findings, as no risk reduction was observed for post‐diagnostic use of this drug group. Nevertheless, the risk decrease by pre‐diagnostic coxib use is intriguing and merits further studies. To our knowledge, no previous studies have estimated pre‐diagnostic coxib use and BCa survival.

ASA is a non‐competitive and irreversible COX inhibitor and seems to be more selective towards the COX‐1 form [7]. COX‐2 is overexpressed in BCa, but COX‐2 inhibition with coxibs does not appear clearly beneficial for BCa outcomes. Therefore, based on our results and previous research, it is reasonable to consider the role of COX‐1 inhibition or simultaneous inhibition of both isoenzymes in different phases of carcinogenesis. To our knowledge there have been relatively few studies on COX‐1 expression in BCa. Hwang et al. [26] have reported COX‐1 overexpression in stromal cells adjacent to BCa. In the light of current research knowledge and the results of this study, the role of COX‐1 inhibition in anticancer effects of NSAIDs and COX‐1 expression in BCa tissue should be further examined.

Our study has several strengths. The reliability of our results is increased by using the national prescription database to assess NSAIDs use. The database provides comprehensive information on medication purchases including duration, dosage and date of purchase. This allowed us to accurately evaluate pre‐ and post‐diagnostic medication use. We were able to mitigate immortal time bias by analyzing post‐diagnostic NSAID use as time dependent exposure. The advantage of register‐based studies is the reduction of memory bias. With surveys, the exact timing of the use can be significantly more difficult to evaluate, and self‐assessment of medication amounts used is challenging. The most significant strength of this study is the availability of data from national BCa screening programs and the identification of BCa cases from the Finnish Cancer Registry covering all BCa diagnosis made in Finland. Similarly, data on the endpoints, BCa‐specific and overall mortality were reliably collected from the nationwide cause of death register. We were also able to obtain information on co‐morbidities from the hospital discharge registry and minimize bias by evaluating their impact on risk estimates.

There are also some limitations. This analysis assumes the amount of prescription drugs to be corresponding with actual consumption which, however, is uncertain. NSAIDs are widely available over‐the‐counter, thus our data on NSAID usage is likely an underestimation of the true exposure, causing bias towards the null. On the other hand, not all prescribed medicines have necessarily been used, which could also lead to an overestimation of the use. We did not have information on indications of NSAID use. Prescription NSAIDs have been likely prescribed for long term use and for intense pain. Thus, users may have had more chronic diseases and comorbidity compared to non‐users. However, median Charlson index did not differ by NSAID use in our cohort. In contrast, ASA is more likely used for preventive purposes and therefore the risk estimates for this drug group may be less affected by confounding by indication. Coxibs entered the market in Finland in 2000 and could not be analyzed for the entire study period. We did not have any information on lifestyle risk factors including smoking, alcohol consumption or physical activity as these were not available from registries. It should also be noted that Finland's population consists mainly of Caucasians, thus, generalizability to other ethnicities is uncertain.

## 5 Conclusion

Post‐diagnostic use of ASA associated with reduced BCa‐specific mortality, distinguishing ASA from other NSAIDs. Clinical trials are required to determine the ideal ASA dose, frequency, and duration for treating BCa.

Pre‐diagnostic NSAID use slightly lowers BCa mortality without dose dependence. The significance of pre‐diagnostic NSAID use as a prognostic factor in BCa warrants further investigation.

## Author Contributions

**M. Malin:** conceptualization‐supporting, data curation‐lead, formal analysis‐lead, writing – original draft‐lead, writing – review and editing‐supporting. **M. Murto:** conceptualization‐supporting, data curation‐supporting, writing – review and editing‐supporting. **O. Arponen:** conceptualization‐supporting, writing – review and editing‐supporting. **A. Siltari:** conceptualization‐supporting, data curation‐equal, writing – review and editing‐supporting. **M. Artama:** data curation‐equal, writing – review and editing‐supporting. **K. Visvanathan:** conceptualization‐supporting, data curation‐equal, methodology‐equal, supervision‐equal, writing – review and editing‐supporting. **T. Murtola:** conceptualization‐lead, data curation‐supporting, formal analysis‐equal, funding acquisition‐lead, methodology‐equal, project administration‐lead, resources‐lead, supervision‐lead, writing – original draft‐supporting, writing – review and editing‐lead. **A. Jukkola:** supervision‐supporting, writing – review and editing‐supporting.

## Conflicts of Interest

The authors declare no conflicts of interest.

## Supporting information


**Data S1.** Supporting Information.

## Data Availability

Data can be accessed with permission from national authority Findata. https://findata.fi/en/.
