# Supplementary material for: Acetyl Salicylic Acid, COX‐2 Inhibitors and Other NSAIDs and Breast Cancer Survival in a Finnish Population‐Based Cohort
Source: Cancer Rep (Hoboken). 2025 Jul 5;8(7):e70271. doi: 10.1002/cnr2.70271 (PMC12228078; doi:10.1002/cnr2.70271)
Supplement: Supplementary file 1 — Data S1. Supporting Information. [file CNR2-8-e70271-s001.docx]

**Supplementary Figure 1**: Risk of breast cancer specific death among NSAID, ASA and coxib users compared to non-users.

HR 1.10

(1.04-1.16)

HR 1.27 (1.22-1.33)

HR 0.78 (0.75-0.81)

HR 0.76 (0.71-0.81)

HR 0.84

(0.73-0.97)

HR 0.92 (0.81-1.03)

*NSAIDs (Non-steroidal anti-inflammatory drugs), ASA (acetylsalicylic acid)

**Supplementary Table 1:** List of NSAIDs* in clinical use in Finland during 1995-2015. Categorizations used in analysis.

| **Analysis on combined NSAID* use** | **Analysis on ASA* use** | **Analysis on coxib use** | **Analysis on non-ASA, non-coxib NSAID use** |
| --- | --- | --- | --- |
| ASA  Mefenamic acid  Tolfenamic acid  Tiaprofenic acid  Ketoprofen  Naproxen  Tenoxicam  Lornoxicam  Piroxicam  Meloxicam  Indometasin  Ibuprofen  Dexibuprofen  Pyrazolone  Sulindac  Diclofenac  Aceclofenac  Nabumetone  Etodolac  Nabumetone  Celecoxib  Valdecoxib  Etoricoxib  Rofecoxib | ASA | Celecoxib  Valdecoxib  Etoricoxib  Rofecoxib | Mefenamic acid  Tolfenamic acid  Ketoprofen  Naproxen  Tenoxicam  Lornoxicam  Piroxicam  Meloxicam  Indometasin  Ibuprofen  Dexibuprofen  Pyrazolone  Sulindac  Diclofenac  Aceclofenac  Nabumetone  Etodolac  Nabumetone |

*****NSAIDs (Non-steroidal anti-inflammatory drugs), ASA (acetylsalicylic acid)

**Supplementary Table 2:** Overall risk of death by NSAID use before diagnosis. Cohort of 73,170 women diagnosed with breast cancer in Finland 1995-2013.

|  | **NSAIDs overall** | | | **ASA** | | | **Coxibs** | | |
| --- | --- | --- | --- | --- | --- | --- | --- | --- | --- |
|  | n of cases/  deaths | HR (95% CI)  _age-adjusted_ | HR (95% CI)  _multivar.-adjusted_ | n of cases/  deaths | HR (95% CI)  _age-adjusted_ | HR (95% CI)  _multivar.-adjusted_ | n of cases/  deaths | HR (95% CI)  _age-adjusted_ | HR (95% CI)  _multivar.-adjusted_ |
| **Medication use** | | | | | | | | | |
| **None** | 17,196/   9,878 | Ref | Ref | 71,428/  21,541 | Ref | Ref | 60,322/  20,106 | Ref | Ref |
| **Any** | 55,974/  12,642 | 0.87 (0.85-0.90) | 0.93 (0.91-0.96) | 1,743/  979 | 1.23 (1.15-1.31) | 1.21 (1.13-1.29) | 12,848/  2,414 | 0.87 (0.84-0.91) | 0.94 (0.90-0.98) |

*Tertile cut points:

- NSAIDs: 1st tertile 0.1-26.2 DDD/year, 2nd tertile 26.3-55 DDD/year, 3rd tertile > 55 DDD/year
- ASA: 1st tertile 0.1-200 DDD/year, 2nd tertile 200.1-400 DDD/year, 3rd tertile > 400 DDD/year
- coxibs: 1st tertile 0.1-20 DDD/year, 2nd tertile 20.1-42 DDD/year, 3rd tertile > 42 DDD/year

**Supplementary Table 3:** Overall risk of death by NSAID use post diagnosis. Cohort of 73,170 women diagnosed with breast cancer in Finland 1995-2013.

|  | **NSAIDs overall** | | | **ASA** | | | **Coxibs** | | |
| --- | --- | --- | --- | --- | --- | --- | --- | --- | --- |
|  | n of deaths/  cases | HR (95% CI) _age-adjusted_ | HR (95% CI) _multivar.-adjusted_ | n of deaths/  cases | HR (95% CI) _age-adjusted_ | HR (95% CI) _multivar.-adjusted_ | n of deaths/  cases | HR (95% CI) _age-adjusted_ | HR (95% CI) _multivar.-adjusted_ |
| **Medication use** | | | | | | | | | |
| **None** | 8,449/  17,631 | Ref | Ref | 21,626/ 71,032 | Ref | Ref | 19,846/  58,162 | Ref | Ref |
| **Any** | 14,071/  55,539 | 0.96  (0.94-0.99) | 1.00  (0.98-1.03) | 894/  2,138 | 1.17  (1,09- 1,25) | 1.18  (1.10-1.26) | 2,674/  15,008 | 0.93  (0,90-0,96) | 0.97  (0.94-1.01) |

*Tertile cut points:

- NSAIDs: 1st tertile 0.1-33 DDD/year, 2nd tertile 33.1-66 DDD/year, 3rd tertile > 66 DDD/year
- ASA: 1st tertile 0.1-183 DDD/year, 2nd tertile 183.1-314 DDD/year, 3rd tertile > 314 DDD/year
- coxibs: 1st tertile 0.1-28 DDD/year, 2nd tertile 28.1-50 DDD/year, 3rd tertile > 50 DDD/year
